# Integrated miRNA-mRNA spatial signature for oral squamous cell carcinoma: a prospective profiling study of Narrow Band Imaging guided resection

**DOI:** 10.1038/s41598-018-19341-x

**Published:** 2018-01-16

**Authors:** Camile S. Farah, Simon A. Fox, Andrew J. Dalley

**Affiliations:** 1 UQ Centre for Clinical Research, The University of Queensland, Herston Qld, 4029 Australia; 2 Australian Centre for Oral Oncology Research & Education, UWA Dental School, University of Western Australia, Nedlands, WA 6009 Australia

## Abstract

Oral squamous cell carcinoma (OSCC) is a common malignancy for which there is poor prognosis and limited therapeutic options. The objective was to identify mRNA targets of dysregulated miRNAs in OSCC using integrated analysis and understand molecular abnormality in surgical margins. We used biopsies along the spatial axis from normal tissue defined by narrow band imaging (NBI) through conventional white light (WL) margins to tumour from 18 patients undergoing surgical resection for OSCC. Overall 119 miRNA and 4794 mRNA were differentially expressed along the adjacent normal tissue to tumour axis. Analysis of miRNA profiles demonstrated the NBI margins were molecularly distinct from both the tumour and WL margin. Integrated analysis identified 193 miRNA-mRNA interactions correlated to the spatial axis of NBI-WL-T. We used cross-validation analysis to derive a spatial interactome signature of OSCC comprising 100 putative miRNA-mRNA interactions between 40 miRNA and 96 mRNA. Bioinformatic analysis suggests that miRNA dysregulation in OSCC may contribute to activation of the oncostatin M, BDNF and TGF-β pathways. Our data demonstrates that surgical margins defined by NBI leave less potentially malignant residual tissue. The miRNA-mRNA interactome provides insight into dysregulated miRNA signalling in OSCC and supports molecular definition of tumour margins.

## Introduction

The success of tumour resection lies in balancing disease removal with healthy tissue retention^1^. Locoregional relapse continues to challenge the surgical management of oral squamous cell carcinoma (OSCC), occurring in 16–20% of cases and stalling survival rate improvement^2–4^. Despite surgeons’ best efforts to define surgical margins away from carcinoma and dysplasia, and confirmation of this by retrospective histopathology, undetected minimal residual disease (MRD) can be left *in-situ*, precipitating relapse^1,3,5^. Yet non-invasive techniques are available that identify potentially malignant perilesional tissue with improved sensitivity and specificity^6^. Recently we reported that use of Narrow Band Imaging for pre-operative OSCC assessment led surgeons to resect visually normal tissue that harboured significant molecular abnormalities without increasing the surgical field unnecessarily, and significantly decreased locoregional recurrence^7^. Similarly, molecular changes indicative of early tumour development have been demonstrated in histologically ‘normal’ surgical margins of tumours from the larynx, pharynx, and oral cavity^8–10^.

MicroRNAs (miRNA or MIR) typically comprise 22 nucleotides with partial sequence homology to regions within mRNA molecules. They evoke post-transcriptional regulation of gene expression by promoting mRNA degredation and function as broad-acting regulatory hubs that may be impaired by mutation^11^. MicroRNA have huge influence on cellular metabolism, proliferation and differentiation, and are key to our understanding of tumorigenesis. Their link to cancer is strengthened by the clustering of miRNA loci within the genome, often at regions that have mutational association with cancer^12^. Although other studies have reported the importance of miRNA in head and neck SCC^13–21^, none have undertaken genome wide integration of microRNA and their target genes’ mRNA expression. The involvement of perilesional molecular abnormalities in MRD can now be studied through detailed assessment of the miRNA-mRNA interactions found at the tumour site compared to molecularly normal surgical margins, taking advantage of NBI to better determine clean surgical margins *in situ*. This coupling of research provides opportunity for more rigorous investigations into the role of miRNA in oral tumorigenesis as drivers of neoplastic transformation.

The broad aim of our study was to characterise and integrate microRNA and mRNA expression patterns in OSCC using tissue samples along a spatial axis extending from the tumour to disease-free normal surgical margins. We prospectively sampled 18 patients with OSCC using biopsies of tumour, adjacent perilesional tissue (conventional surgical margin), and distant (disease-free) tissue determined by NBI. Firstly, we sought to use molecular analysis of miRNA and mRNA to confirm and extend our previous findings that conventionally determined surgical margins can harbour molecular abnormality. We characterised this spatial gradient of dysregulation at the molecular level and through pathway and gene ontology analysis. As a consequence we aimed to create a miRNA profile of OSCC based upon improved definition of adjacent normal (non-diseased) tissue controls using NBI defined margins as opposed to the currently accepted delineation under white light. The other major objective of our study was to integrate the miRNA and mRNA data in order to select and validate an miRNA-mRNA signature for OSCC. Through this and analysis of ontology and pathways we aimed to derive biological insight into the role of miRNA in the perturbation of signalling in oral tumorigenesis.

## Results

### Clinical observations

Full patient demographic and clinical details have already been published in tabular form together with photographs that describe the positioning of sample biopsies^7^. Briefly, patient age, sex and patterns of environmental risk factor exposure (tobacco and alcohol intake) were typical for the RBWH Head and Neck Cancer clinic. All tumour biopsies were negative for human papilloma virus (HPV)^7^. Tumour histology was predominantly moderate (n = 10) to well (n = 7) differentiated OSCC, with 1 case of verrucous carcinoma^7^. The most current follow-up data (up to 7 years post-surgery) shows that of the 18 patients, 1 patient (5.55%) declined follow-up. At 5 years follow-up, 14/17 patients (82.35%) were alive with no local recurrence, 1/17 patient (5.88%) had died from their disease, and 2 patients (11.76%) had died disease-free from other causes. In total, 16/17 patients (94.11%) who were followed for a minimum of 5 years were still alive and had not developed local recurrence.

### Processing of miRNA and mRNA microarray data

**Figure 1:**
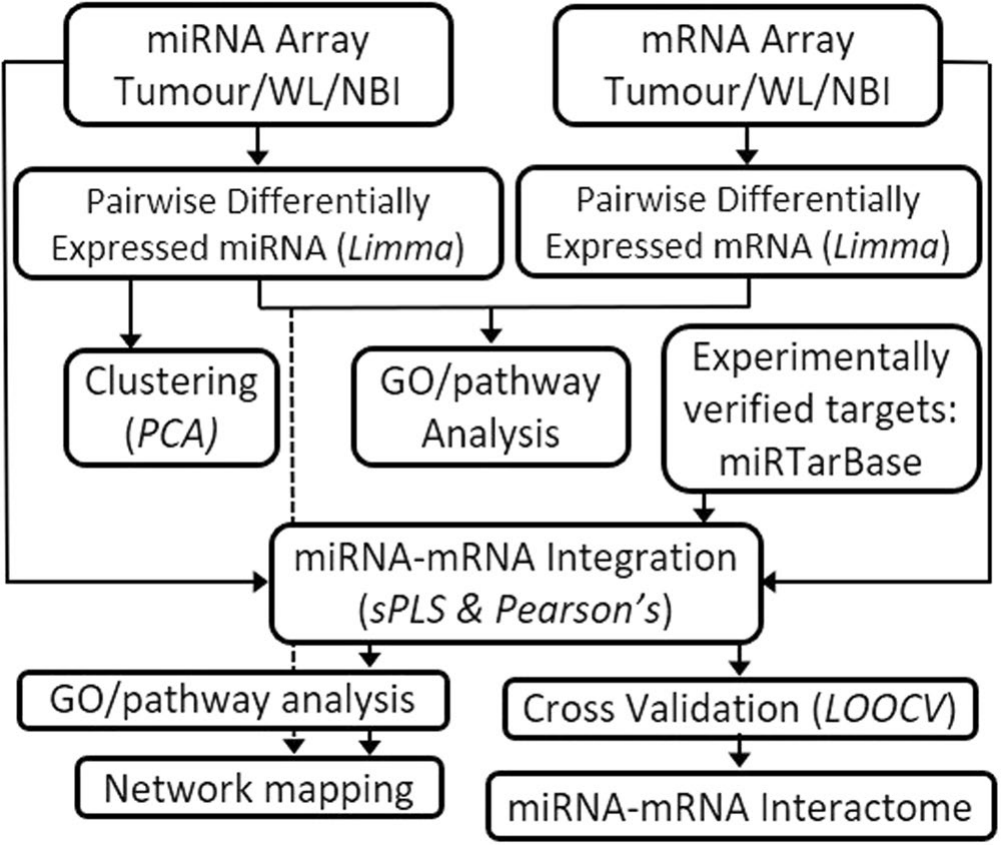
Bioinformatics pipeline.

GeneChip® Human Genome U133 Plus 2.0 Arrays provided 54675 mRNA probe sets, while SurePrint® G3 Human miRNA Microarrays (Release 16) provided 1205 human miRNA probes and 144 human-viral miRNA probes (not reported here). Whole genome mRNA and miRNA expression data from 54 Affymetrix mRNA arrays and 54 Agilent miRNA arrays were processed. Each dataset comprised 3 tissue samples, T, WL and NBI, from each of 18 patients. Quality control procedures eliminated data from 5 mRNA arrays and 10 miRNA arrays (Table 1). After normalisation and preliminary filtering, 38989 mRNA probes and 458 miRNA probes entered the bioinformatics pipeline (Fig. 1).

**Table 1:** Quality control filtering and omission of mRNA or miRNA array data.

	Biopsy	Patient Number*																	
	Site	01	02	03	04	05	06	07	08	09	10	11	12	13	14	15	16	17	18
mRNA	T	1	1	1	1	1	1	1	1	1	1	1	1	1	1	1	1	1	1
	WL	1	1	1	1	1	1	1	1	1	1	1	1	1	1	1	1	1	1
	NBI	1	1	1	1	0	0	0	1	1	1	1	0	0	1	1	1	1	1
miRNA	T	1	1	1	1	1	1	1	1	1	1	1	1	0	1	1	1	1	1
	WL	1	1	1	1	1	1	1	1	1	1	1	1	1	0	1	1	1	1
	NBI	1	1	1	1	1	0	0	1	0	1	0	1	1	1	0	0	0	0

1 = data included, 0 = data excluded. *Corresponds to Farah *et al*.^7^.

### miRNA expression profiles in OSCC margins

**Figure 2:**
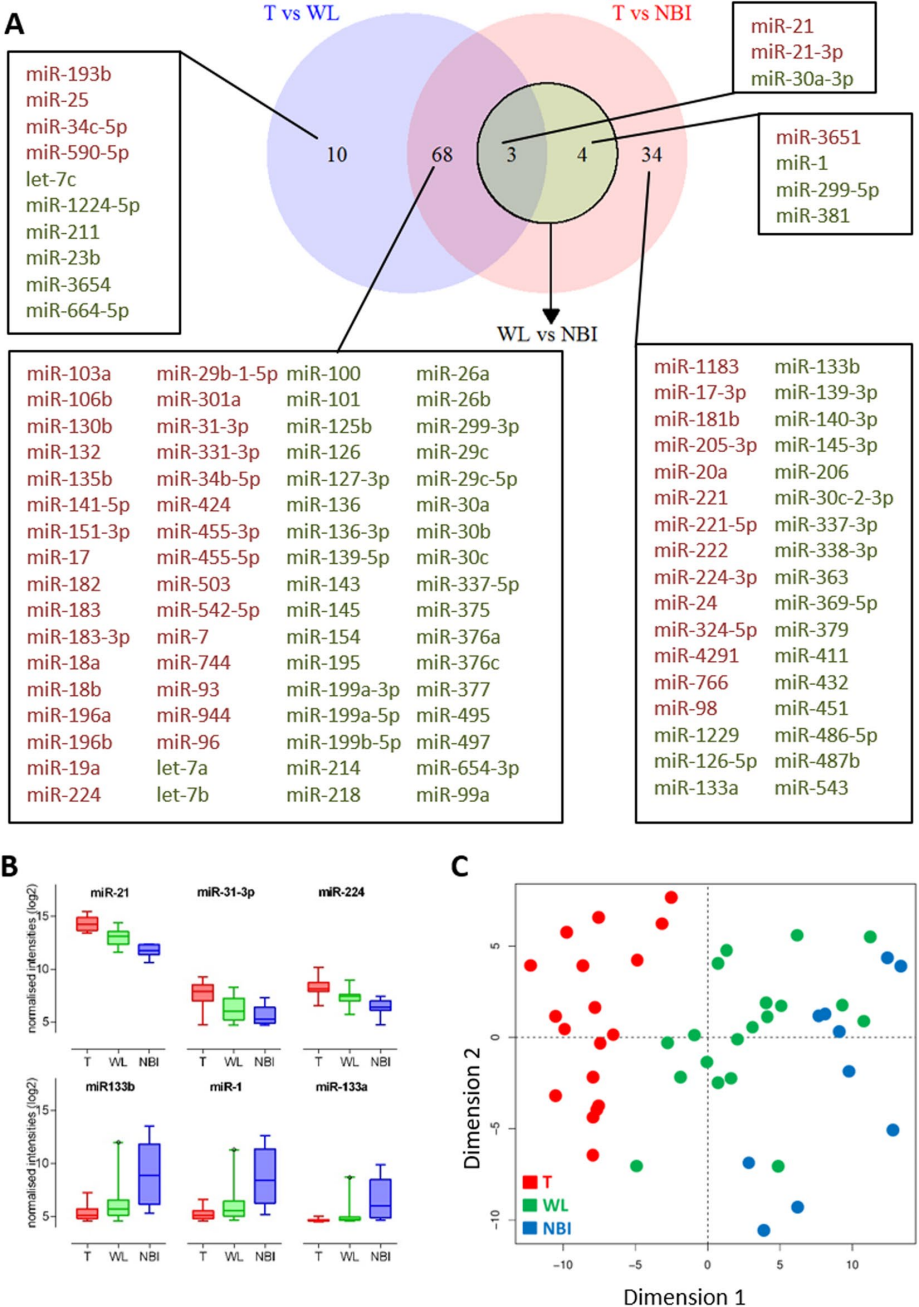
Differential miRNA expression in OSCC margins. (**A**) Venn diagram illustrating the relationship between differentially expressed miRNAs in the three discrete comparisons: T vs WL (Blue Circle), T vs NBI (Red Circle), WL vs NBI (Green Circle). For each group, upregulated miRNAs (red) and downregulated miRNAs (green) are tabulated. (**B**) Boxplots showing the expression profiles across the tumour margins of the 3 most upregulated and downregulated miRNA derived from the T vs NBI comparison. (**C**) Principal Component Analysis (PCA) plot of miRNA differential expression data showing spatial segregation according to biopsy location: T- tumour; WL – white light; NBI – narrow band imaging.

**Figure 3:**
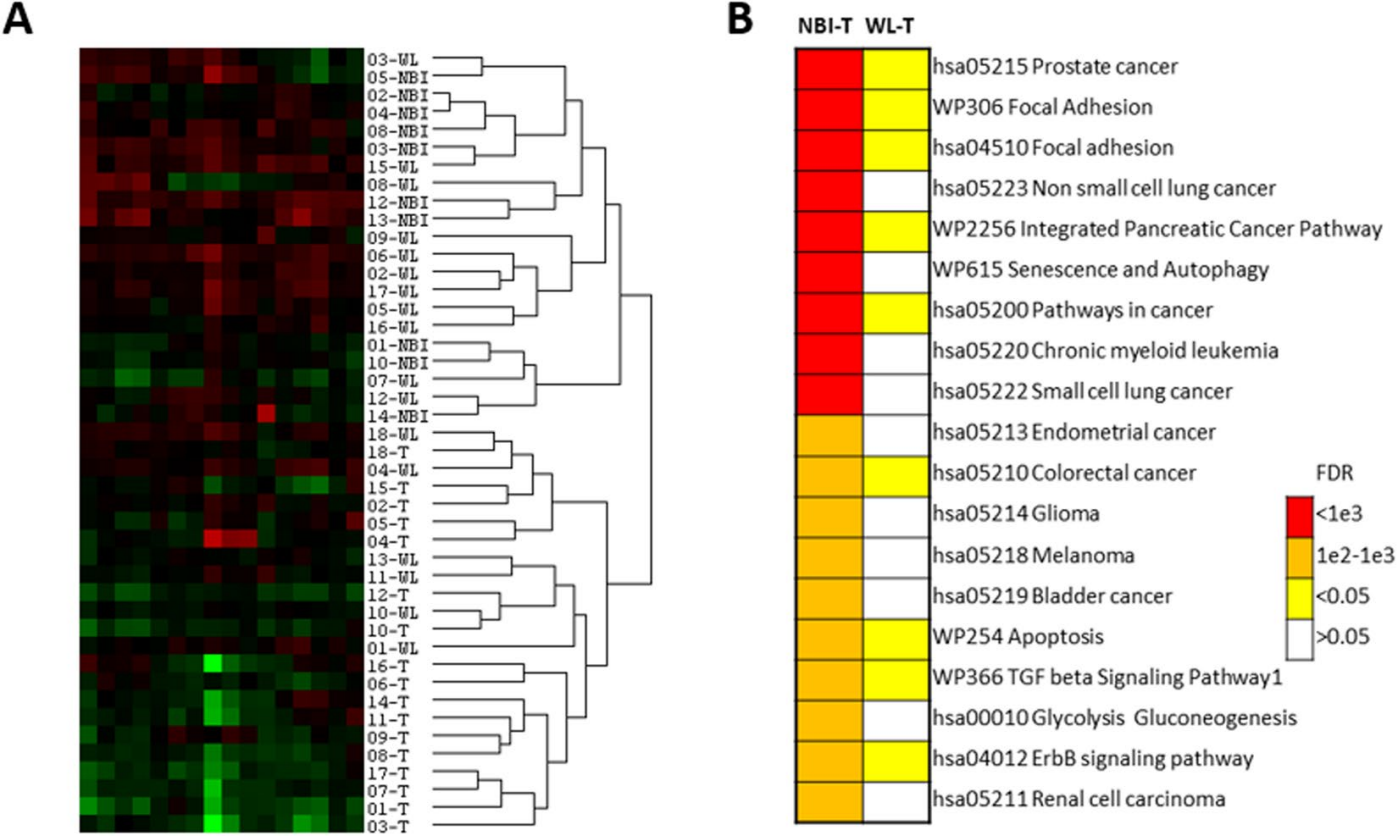
(**A**) Unsupervised hierarchical clustering of differentially expressed miRNA and abridged heatmap. Annotations are according to patient number and biopsy site. (**B**) Pathway analysis by gene set enrichment of dysregulated miRNAs in OSCC margins. Pathways were derived from the KEGG (hsa) and Wikipathways (WP) databases. Analysis was performed separately for the T vs NBI and T vs WL comparisons and the level of significance is indicated by different colours.

We investigated the miRNA expression pattern by comparisons across the tumour, WL and NBI defined margins. Specifically, we were interested in differences in molecular dysregulation across the tumour borders as defined by either WL or NBI and whether there was molecular distinction between the margins. We observed 137 miRNA probes (corresponding to 119 miRNA genes) were differentially expressed in at least one sample site (Fig. 2A). Overall the largest number of statistically significant differentially expressed miRNA were found in the T vs NBI comparison. Although the majority of these miRNA were common to both the T vs WL and T vs NBI comparison, there were still 34 which were unique to T vs NBI. The normalised expression profile of the most differentially up and down regulated miRNAs in the T vs NBI comparison are shown in Fig. 2B. To further investigate whether miRNA expression profiles could differentiate the tumour margins we used principal component analysis (PCA) to identify a discriminatory component that separated tumour from the WL and NBI samples (Fig. 2C). Whilst PCA did not fully segregate WL from NBI samples, the PCA plot placed most WL samples between NBI and tumour samples (Fig. 2C), therefore the discriminatory component segregated tumour, WL and NBI tissue biopsies appropriately along the adjacent normal tissue to tumour axis based solely upon their miRNA profiles, in a similar pattern to that described by us previously for mRNA^7^. Hierarchical clustering of the miRNA data was consistent with this finding with some WL samples in the same major cluster as the T samples (Fig. 3A). Given dysregulation of miRNA across the OSCC margins, we used statistical set enrichment analysis to understand the pathways which the dysregulated miRNAs participate using miEAA. We independently analysed the T vs WL and T vs NBI differentially expressed microRNAs for enrichment of miRNAs associated with specific pathways. Overall there were 28 pathways for T vs WL and 127 for T vs NBI which we calculated were significantly enriched (FDR < 0.05), the top 20 (ranked by FDR) are shown in Fig. 3B. Generally, the significantly enriched pathways comprised those associated with cancer and cancer associated signalling. Consistent with our other analysis, there was a greater level of pathway dysregulation across the spatial axis NBI-T than for WL-T (Fig. 3B).

### mRNA expression profiles in OSCC margins

**Figure 4:**
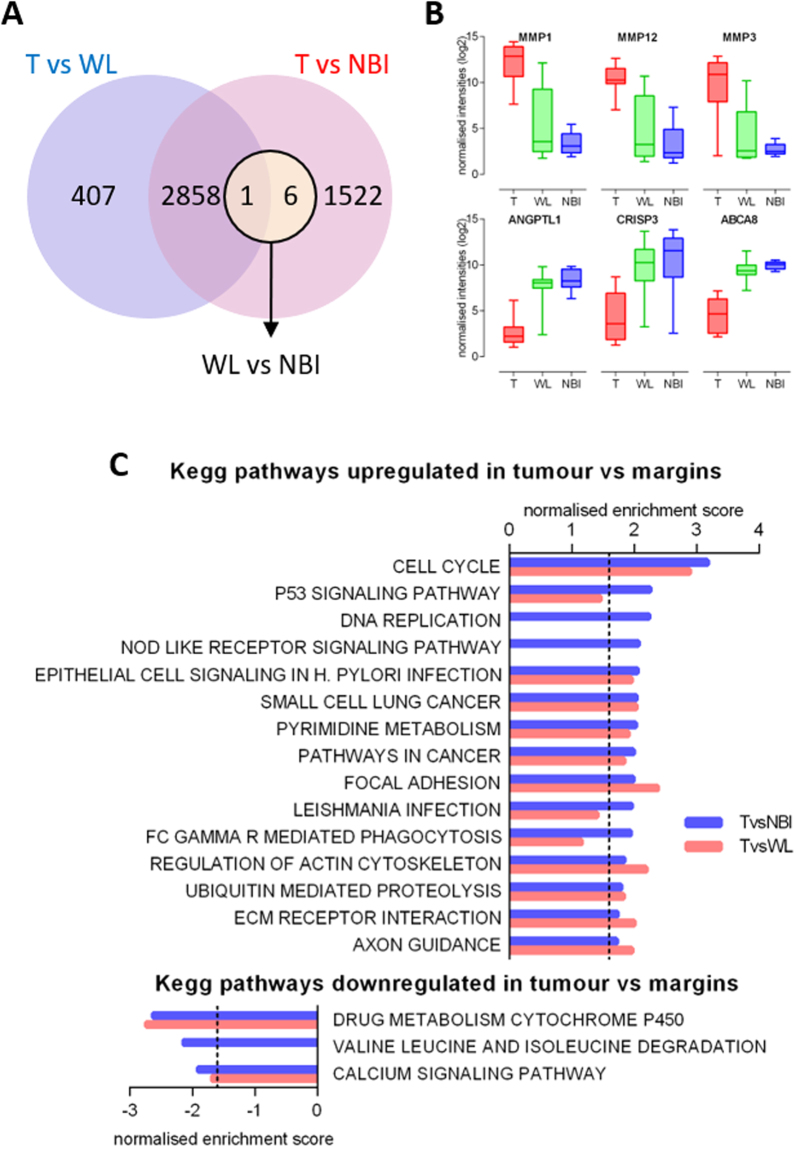
Differential mRNA expression in OSCC margins (**A**) Venn diagram illustrating the relationship between differentially expressed mRNAs in the three discrete comparisons: T vs WL (Blue Circle), T vs NBI (Red Circle), WL vs NBI (Green Circle). (**B**) Boxplots showing the expression profiles across the tumour margins of the 3 most upregulated and downregulated mRNA derived from the T vs NBI comparison. (**C**) Pathway analysis of dysregulated genes (mRNA) between the OSCC margins. Up and down regulated pathways were determined using GSEA and the MsigDb curated KEGG database genesets. Pathways are ranked according to normalised enrichment score generated by GSEA. Threshold of significance is shown by the dashed line.

We have previously reported our initial analysis of the differentially expressed mRNA dataset for OSCC margins by hierarchical clustering and PCA analysis to demonstrate molecular divergence between tumour, WL and NBI defined margins^7^. Consistent with the microRNA analysis, the largest number of statistically significant differentially expressed genes were found in the T vs NBI comparison (Fig. 4A) and there were very few genes identified by this analysis as significantly differentially expressed (DE) between WL and NBI. While the majority of the DE genes were common to both the T vs WL and T vs NBI comparisons, there were substantial numbers of genes unique to the individual comparisons (Fig. 4A). The normalised expression profile of the most up and down regulated mRNAs in the T vs NBI comparison are shown in Fig. 4B. To predict the biological implications of these DE genes and further elucidate the molecular differences between the tumour margins, we performed pathway analysis using the GSEA platform for pathway enrichment. We interrogated the KEGG pathway gene sets curated in MSigDB using ranked DE gene lists from the T vs NBI and T vs WL comparisons (Fig. 4C). Our analysis found that there was a preponderance of upregulated pathways in comparison to down regulated pathways (Fig. 4C). Furthermore, 5 of the top 15 significantly enriched upregulated pathways in the T vs NBI comparison were not significant for T vs WL. Interestingly, although there were few significantly DE genes in the WL vs NBI comparison, gene ontology analysis showed they were highly enriched for genes involved in oxygen transport (GO:0015671 Biological Process Padj = 1.6E-09). The dysregulation of mRNA expression along the NBI to T spatial axis is also demonstrated by the normalised expression profile of the most up and down regulated mRNAs in the T vs NBI comparison (Fig. 4B).

### Integration of miRNA-mRNA datasets

In order to integrate miRNA and mRNA expression profiles we first used significantly DE miRNA then combined this data with experimentally verified interactions from miRTarBase^22^. Of the 119 differentially expressed miRNA in at least one pairwise comparison, 92 had experimentally verified targets on the miRTarBase database^22^. Sparse Partial Least-Square (sPLS) analysis of median expression values across the biopsy sites (T, WL and NBI) identified 45 miRNA that were supported by a total of 193 target (mRNA) to effector (miRNA) correlations that were statistically significant (FDR < 0.05). The WL vs NBI paired comparison did not generate miRNA-mRNA associations (data not shown).

**Figure 5:**
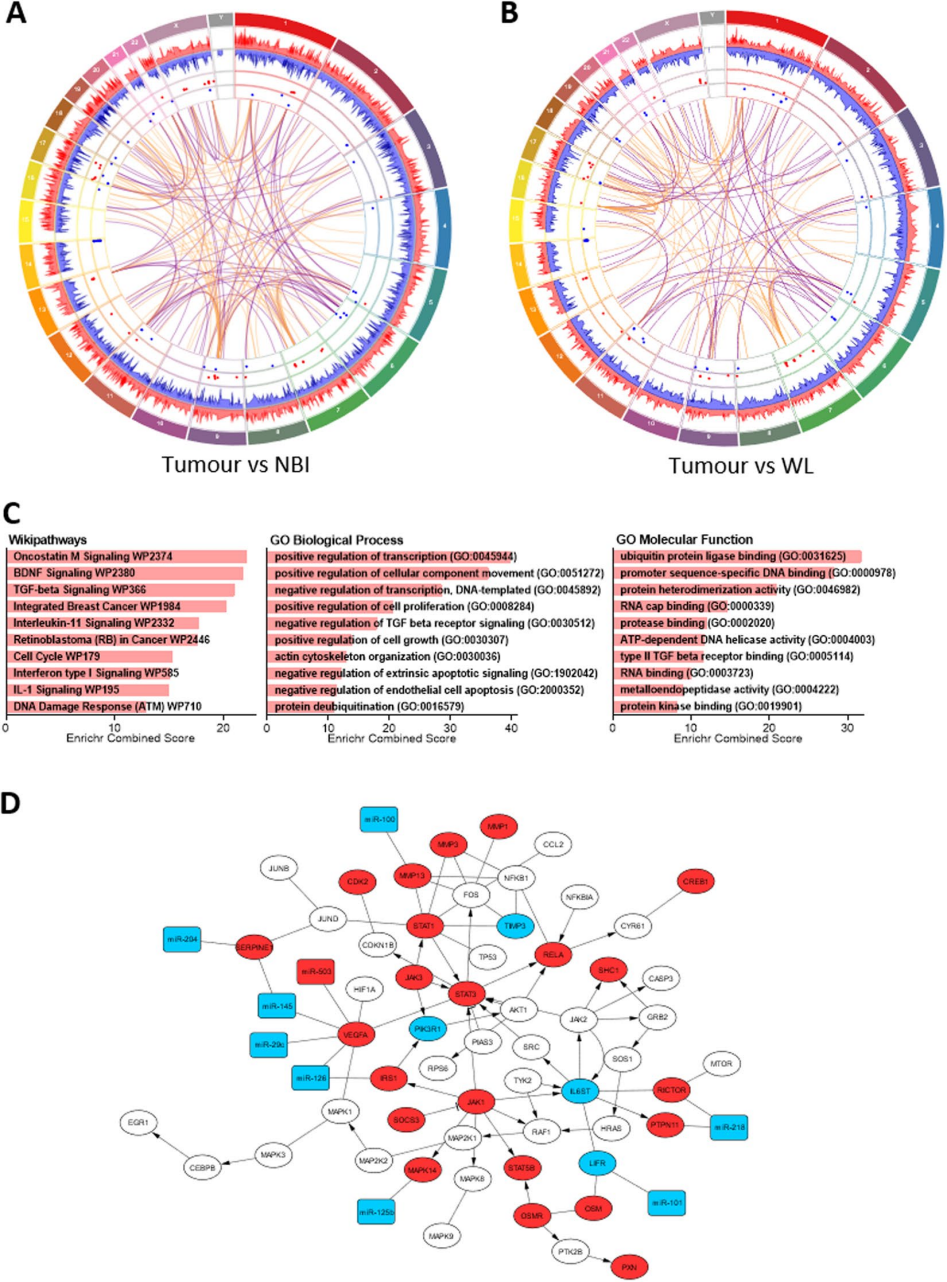
Circos plots of gene and miRNA differential expression and potential regulatory linkages for (**A**) tumour vs NBI and (**B**) tumour vs WL. Outermost track is chromosome mapping. Histogram plot tracks show differential gene (mRNA) expression as up (red) and down (blue) regulated (−Log(10) P-value). Dot plot tracks show differential microRNA (miRNA) expression as up (red) and down (blue) regulated (−Log(10) P-value). Innermost track (centre) shows correlated miRNA-mRNA linkages as reciprocal (purple) and non-reciprocal (orange). (**C**) Functional enrichment of the correlated miRNA-mRNA dataset by overrepresentation analysis of pathways and gene ontology. The target correlated mRNA were used with the EnrichR analysis tool to derive significantly perturbed pathways and gene ontology associations. The top 10 pathways/ontology terms for each analysis are shown ranked by the EnrichR combined score parameter. (**D**) Functional relationships of candidate miRNA-mRNA interactions mapped onto the Oncostatin M signalling network using Cytoscape. mRNA nodes are oval and miRNA nodes are rectangular. For both mRNA and miRNA upregulated nodes are red and downregulated are blue.

The differential expression data (fold-change and adjusted p-value) for the pair-wise comparisons, T vs WL and T vs NBI, were incorporated with the integrated miRNA-mRNA data. We used Circos plots to integrate the data from the miRNA and mRNA differential expression datasets, the miRNA-mRNA interactions and chromosomal mapping of individual targets as shown in Fig. 5A and B. Greater molecular divergence was evident in the T vs NBI comparison than the T vs WL comparison (Fig. 5A and B respectively). In total there were 139 putative miRNA-mRNA linkages within the T vs NBI paired comparison (86 common and 53 unique), in contrast there were 117 putative miRNA-mRNA linkages in the T vs WL paired comparison (86 common and 31 unique). There were 19 statistically significant additional miRNA-mRNA correlations which did not map to the individual pairwise DE comparisons but were identified by spatial correlation along the tumour to normal tissue axis. This spatial integration included two miRNAs (miR-181a and miR-204) and six additional mRNA (HMGA1, KDM4A, MRPL38, RNF24, SLAIN1, PLEKHA8) that were not identified by individual paired comparisons (T vs WL; T vs NBI). To better understand which biological functions and pathway perturbations are associated with dysregulated miRNA-mRNA interactions in OSCC we applied functional and pathway annotations using overrepresentation analysis with the EnrichR analysis tool (Fig. 5C). The list of target mRNAs from the correlated dataset was used to explore for GO annotation terms (from GO consortium) and biological pathways based upon the curated Wikipathways database. There were 63 GO biological process (BP) terms significantly (Padj < 0.05) associated with the dataset. These terms were summarised using REVIGO (similarity = 0.5) to reduce redundancy and the top 10 BP terms are shown in Fig. 5C with transcription regulation, cellular movement and proliferative signalling predominating. There were 16 GO molecular function (MF) terms associated with the dataset (Padj < 0.05) with the top 10 following consolidation using REVIGO shown in Fig. 5C with terms associated with ubiquitination and transcription regulation enriched. As shown in Fig. 5C overrepresentation analysis of pathways from the Wikipathways database in the dataset was consistent with perturbation of pathways implicated in tumorigenesis. In order to further explore and illustrate dysregulated miRNA-mRNA signalling we imported the highest ranked Oncostatin M signalling pathway into Cytoscape using the Wikipathways app, visualised it as a network and mapped the differentially expressed mRNAs, the correlated miRNAs and their differential expression (Fig. 5D). This network included the miR-204/SERPINE1 interaction identified through spatial correlation. Correlated miRNA-mRNA interactions in this pathway involved key molecules implicated in carcinogenesis such as JAK/STAT pathway members, MMP13 and the protein tyrosine phosphatase PTPN11.

### Development and validation of an integrated miRNA-mRNA signature of OSCC margins

**Figure 6:**
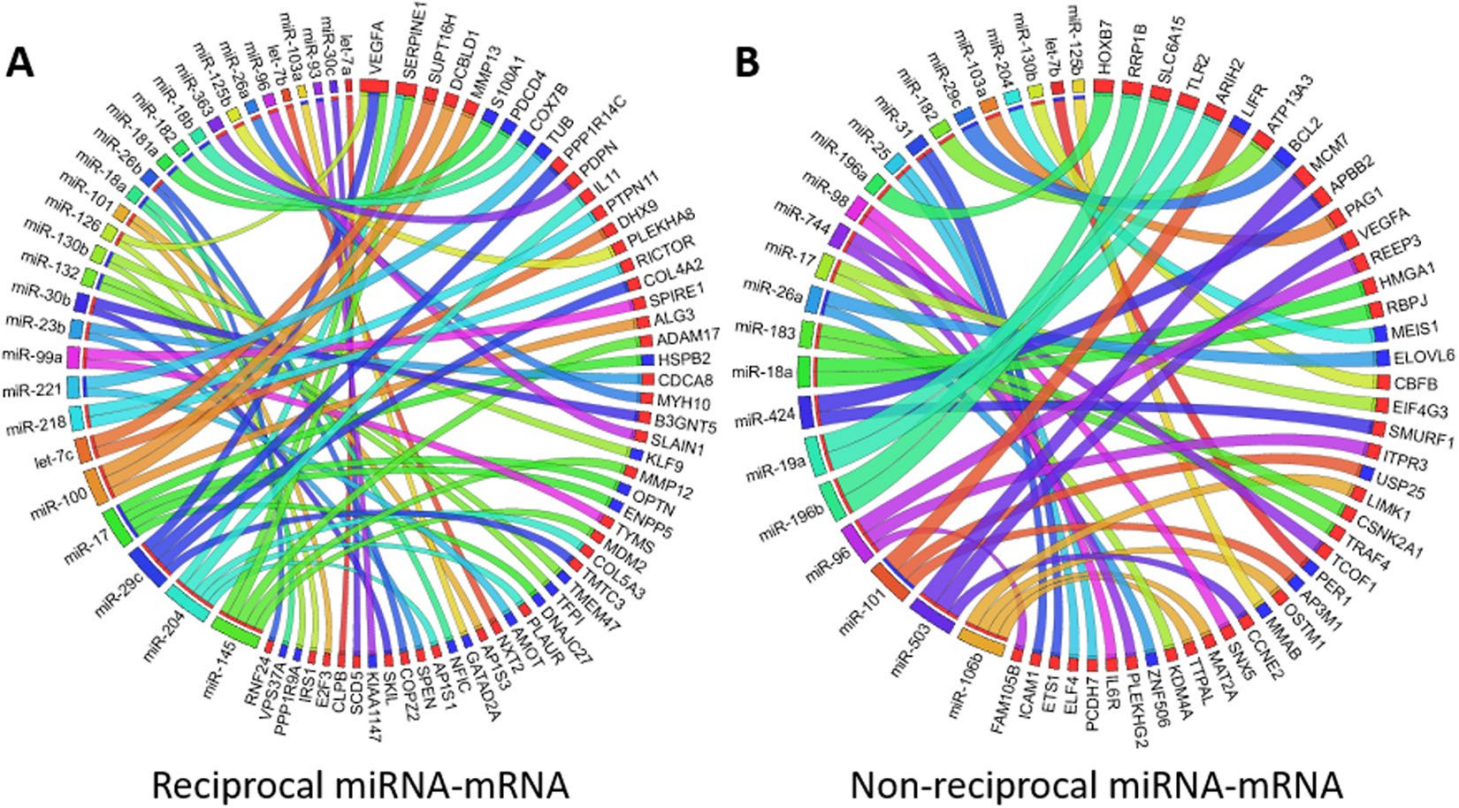
Circos plots of the integrated miRNA-mRNA spatial signature showing (**A**) reciprocal and (**B**) non-reciprocal miRNA-mRNA linkages. Data includes all interactions which were statistically robust (cross validation stability >0.5) and spatially correlated with the normal tissue to tumour axis (Pearson’s correlation FDR adjusted p < 0.05). Ribbon colour indicates the originating miRNA. Ribbon thickness is proportional to the stability of the interaction in the cross-validation analysis (0.5–1). The colour of the tabs adjacent to the gene names indicates up (red) or down (blue) mRNA expression in the tumour.

We next undertook additional analysis to develop and validate an integrated molecular signature of OSCC margins derived from the correlated miRNA-mRNA interactome shown in Fig. 5A and B. We took the integrated dataset which was modelled against the adjacent normal tissue to tumour spatial axis given by the three biopsy sites (NBI-WL-T) and applied a statistical cross validation test. As described above we were able to capture interactions which were not significant within the context of pairwise comparison but might be significant when considered along the entire spatial axis. The statistical robustness of each miRNA-mRNA pairing was assessed by cross validation (LOOCV) and filtering by threshold of >0.5 (>50% of data supportive) prior to inclusion in the signature. From a total of 190 statistically significant miRNA-mRNA interactions identified by the initial sPLS and Pearson’s correlation analysis, 100 were subsequently validated by LOOCV (stability) involving 40 miRNAs and 96 genes. The overall spatially integrated signature following LOOCV filtering is shown in Fig. 6 for miRNA-mRNA interactions grouped according to reciprocal (Fig. 6A) and non-reciprocal (Fig. 6B) correlations (Additional file 1). Of the 100 interactions, 13 were the additional interactions derived uniquely by spatial correlation and absent from the pairwise comparisons (Additional File 1). Two mRNA, VEGFA (miR-126, miR-145, and miR29c) and SERPINE1 (miR-145 and miR-204) were subject to regulation by more than one miRNA. The integrated miRNA-mRNA signature included 5 unique miRNA (miR-744, miR-18b, miR-98, miR-221 and let-7b) not previously reported in OSCC microRNA profiling^23^.

## Discussion

This study of 18 patients with intra-oral OSCC used both miRNA and mRNA expression profiling and bioinformatic analysis to evaluate the molecular divergence between tumour core and adjacent tissue judged negative for tumour using either conventional white light or Narrow Band Imaging. The miRNA expression profile alone provided unambiguous evidence that the surgical margins determined by NBI possess fewer molecular abnormalities than the more conservative surgical margins determined by WL examination. Pathway analysis of both the mRNA and miRNA dysregulated datasets also demonstrated greater level of biological distinction between NBI and tumour than for WL and tumour. These observations concur with our prior publication which examined mRNA expression profiles alone in the same patient cohort^7^, and strongly supports our premise that resection to surgical margins determined by NBI rather than by WL examination leave less potentially malignant residual tissue and thereby increase surgical success and decrease locoregional recurrence.

Microscopic tumour at surgical margins has been shown to increase 5-year mortality rates by 90% in a cohort of 707 intra-oral OSCC patients for whom 14.6% of surgical resections produced involved margins^24^. The reported accuracy of post-operative histopathology exceeds 95%^25^, yet around 30% of patients with histologically negative margins experience relapse, prompting concerns about its prognostic sensitivity^5,26–28^. Underlying local relapse in patients with clear surgical margins are two issues: minimal residual disease (MRD) and second primary tumour development from a field of pre-neoplastic cells^1^. By design, this study provides insight into both of these disease processes, but is primarily focused on the avoidance of MRD. Defining the dysregulation of biological signalling networks across the tumour margins enables the development of a more precise molecular definition of an effective margin.

The three biopsy sites studied (NBI, WL, T) comprise a spatial axis from disease-free histologically normal tissue, through non-cancerous tissue bearing evidence of abnormality, to tumour core. Initially, all of the miRNA and mRNA differential expression data was modelled collectively against this spatial axis and combined to form a putative miRNA-mRNA interactome. We strengthened our analysis by considering only experimentally validated miRNA-mRNA interactions in order to develop a more robust and less complex integrated signature but this approach is necessarily limited since many putative interactions are as yet unverified. This spatially referenced interactome is a “proper superset” of the three discrete paired comparisons (T vs NBI, T vs WL, WL vs NBI), meaning that it includes miRNA-mRNA interactions that do not occur in any of the paired comparisons. Subsequent analysis identified which of the statistically robust miRNA-mRNA interactions were operative in each paired comparison, and could be correlated with the normal tissue – tumour spatial axis.

Integration of the putative miRNA-mRNA interactome with the paired comparisons of T vs WL and T vs NBI generated a complicated picture of miRNA-mRNA interaction (Fig. 5A and B) which was further refined by cross validation. Throughout this analysis we consistently found more miRNA-mRNA interactions in the TvsNBI comparison than the TvsWL comparison and a gradient of miRNA-mRNA dysregulation along the spatial axis. Together with the ideographic representations (Figs 5 and 6), the present study is the most comprehensive analysis of putative miRNA-mRNA interactions within OSCC published to date. Furthermore, these findings have broader implications since there is increasing interest in molecular heterogeneity within tumours and their boundaries^29,30^ although few studies have examined tumour margins spatially to the extent of integrated molecular detail used here.

As described, our integrated miRNA-mRNA signature included 5 unique miRNA (miR-744, miR-18b, miR-98, miR-221, let-7a and let-7b) not previously reported in OSCC or head and neck squamous cell carcinoma (HNSCC) microRNA profiling^23^. Based upon recent comprehensive review of aberrant microRNAs in HNSCC^23^ of the 40 microRNA found in the integrated signature reported here 28 miRNA had been previously reported as dysregulated in OSCC^31–34^ and 7 miRNA had been reported in HNSCC^23^. The direction of dysregulation found in our own study was consistent with those reported for these miRNAs elsewhere. Thus, the miRNA profile we have developed through integrated analysis is both a consolidation and extension of these previous studies.

Enrichment analysis of dysregulation in the individual miRNA and mRNA data as well as the integrated miRNA-mRNA dataset provided further insight into the mechanisms and downstream consequences of this dysregulated signalling. Broadly, pathway enrichment of the miRNA and mRNA DE data was consistent with the findings of our study supporting increasing molecular dysregulation along the NBI to tumour axis. Focal adhesion was amongst the top 10 dysregulated pathways in both the miRNA and mRNA DE datasets (Figs 3B and 4C) but was not identified in the integrated data. In the integrated data GO analysis demonstrated a preponderance of dysregulation in gene regulatory molecular function, a well-recognised feature of miRNA in preferentially targeting nuclear signalling mechanisms^35^. Key oncogenic pathways were identified as perturbed across OSCC margins including oncostatin M, brain derived neurotrophic factor (BDNF) and TGF-β signalling pathways (Fig. 5C). Oncostatin M signalling has been previously demonstrated to contribute to tumour progression and metastases through JAK-STAT signalling^36,37^. Aberrant expression of oncostatin M has been described in OSCC and cervical SCC as associated with poor clinical outcomes^38,39^. In oral and cervical SCC tissue transglutaminase (TGM2) has been identified as an important downstream mediator of Oncostatin M signalling^40^, and in our own data we found that TGM2 was upregulated in both T vs NBI and T vs WL. The oncostatin M, BDNF and TGF-β pathways have been demonstrated to co-operate to drive EMT and cancer stem cell expression signatures in other cancers. Recent evidence indicates that oncostatin M cooperates with TGF-β signalling to activate EMT via SMAD3^41^. In our own data SMAD3 ranked highest when we used Enrichr to interrogate the database of transcription factor targets (TRANSFAC) with the integrated dataset (results not shown). Similarly in non-small cell lung cancer, STAT3 and BDNF signalling have been shown to cooperatively activate proliferative signalling^42^. While the contribution of STAT3 signalling to HNSCC progression are well recognised, the mechanisms underlying this activation are poorly understood^37^. Our data support a contribution by miRNA and their targets to this dysregulation with downstream effects upon multiple interacting oncogenic pathways. The ultimately derived putative miRNA-mRNA signature included interactions that had statistically supported correlation to the normal tissue–tumour spatial axis, as well as excluding all non-statistically robust interactions. Thus, the spatially correlated interactome remains a “proper superset” of the three discrete paired comparisons. Out of 100 interactions, it contains 13 which were exclusively identified by spatial integration including two miRNAs (miR-181a and miR-204) and five additional mRNA (HMGA1, KDM4A, RNF24, SLAIN1, PLEKHA8) that were not represented within the individual paired comparisons (T vs WL; T vs NBI). These miRNA-mRNA interactions require further investigation but this supports the validity of our underlying premise that we could exploit the availability of the three biopsy locations to derive greater information regarding miRNA and mRNA dysregulation using spatial analysis.

In this study we observed a large number of non-reciprocal miRNA-mRNA correlations which is intuitively inconsistent with our current understanding of miRNA mechanisms of regulation^11^, but is a common finding in integrated analysis of miRNA-mRNA expression profiles^43,44^. Recent evidence suggests that such positive correlations may be a consequence of an adaptive response to changes in mRNA expression and reflect homeostatic mechanisms regulating miRNA expression^44^. Furthermore, we included non-reciprocal correlations in our network analysis because miRNA interactions with their targets may depend upon the topology of the signalling pathway^45^. For this reason, while we focussed upon negative correlations since they reflect direct miRNA regulatory networks, we did not entirely exclude non-reciprocal correlations from our analysis.

## Conclusions

This study has shown that miRNA differential expression, putative miRNA-mRNA interactions and dysregulated biological signalling are greater in T vs NBI than in T vs WL. Our analysis across the three biopsy sites strongly supports our premise that resection of OSCC to surgical margins that are determined by NBI rather than by WL will leave less potentially malignant residual tissue and thereby increase the likelihood of surgical success. This is significant for surgical management of oral cancer but also has implications for molecular analyses of cancer genetic dysregulation using conventionally defined “normal” tissue observed under white light visualization. By stringent analysis we identified the specific miRNA-mRNA interactions that correlated with the normal tissue to tumour spatial axis. This signature is robust, statistically validated and is not conflicted by control “normal” tissue which may harbour molecular abnormality. These particular interactions are likely to include miRNA-mRNA interactions that accompany oral neoplastic transformation. Our integrated bioinformatic analysis suggests that miRNA dysregulation in OSCC could be a mechanism contributing to activation of the oncostatin M, BDNF and TGF-β pathways with signalling via STAT3 and SMAD3. Finally, our statistically robust miRNA signature in OSCC is a foundation for translational development of molecular assays which can be applied to the definition of surgical margins with greater fidelity than current methods.

## Methods

### Patient group

Eighteen patients with intra-oral squamous cell carcinoma (excluding lip, pharynx and hypopharynx) who were scheduled for surgical resection were enrolled prospectively. Patient demographics, tumour characteristics and surgery have been described by us previously^7^. This study was run in strict accordance with the principles outlined in the Declaration of Helsinki (2008) after ethical review and approval from the Royal Brisbane and Women’s Hospital Human Research Ethics Committee (HREC/08/QRBW20 and HREC/10/QRBW336). All subjects provided written informed consent to participation in the study.

### Sample collection and characteristics

Prior to surgery, primary OSCC sites were visualised under White Light (WL) and then by Narrow Band Imaging (NBI) using an Olympus NBI ENF-VQ nasendoscope with CLV-180 light source and processor (Olympus Medical Systems Corp., Tokyo, Japan). Upon completion of the OSCC resection which was taken to ≥5 mm beyond the NBI defined surgical margin, a 4 mm punch biopsy was taken for analysis from within the following zones as reported by us previously^7^.

1. The NBI margin – 5 mm beyond the limit of tissue abnormality visible by NBI.
2. The White Light (WL) margin –5 mm beyond the limit of tissue abnormality visible by White Light.
3. The core of the primary tumour (T).

The biopsies were immediately immersed in RNA*later* (Ambion, Life Technologies, Carlsbad, USA) and subsequently stored at −80 °C. The NBI margin sample is histologically normal disease-free tissue adjacent tumour as previously reported by us^7^.

### RNA isolation

Frozen biopsies were ground in liquid nitrogen then incubated in 500 μL of lysis buffer (Buffer RLT; Qiagen, Hilden, Germany), with 200 ng of Proteinase K (Invitrogen, Life Technologies, Carlsbad, USA) overnight with mixing at 37 °C. After incubation, 200 μL of lysate was used for RNA isolation with TRIzol Reagent (Invitrogen, Life Technologies, Carlsbad, USA) following the manufacturer’s protocol that was optimised to increase nucleic acid recovery by addition of 10 μg of RNase-free glycogen as a carrier and an overnight incubation at −20 °C. After DNase treatment with the TURBO DNA-*free* Kit (Ambion, Life Technologies, Carlsbad, USA), RNA was purified by sodium acetate precipitation (Ambion, Life Technologies, Carlsbad, USA); quality and quantity assessments used a NanoDrop spectrophotometer (Thermo Fisher Scientific, Waltham, USA) and Qubit fluorometer (Invitrogen, Life Technologies, Carlsbad, USA). RNA integrity was assessed using an Agilent 2100 Bioanalyzer and RNA 6000 Nano kit (Agilent Technologies, Santa Clara, USA).

### Gene expression profiling and bioinformatic quality control

This study utilised GeneChip® Human Genome U133 Plus 2.0 Arrays (Affymetrix, Santa Clara, USA) and SurePrint® G3 Human miRNA Microarrays Release 16, 8 × 60 K (Agilent Technologies, Santa Clara, USA) to derive whole genome mRNA and miRNA expression data from three samples (NBI, WL, and T) for each of 18 patients, giving a total of 108 expression profiles. 100 ng of total RNA was the input for target preparation for both mRNA and miRNA hybridisations using the GeneChip 3′ IVT Express Kit (Affymetrix, Santa Clara, USA) and the miRNA Microarray System with miRNA Complete Labeling and Hyb Kit (Agilent Technologies, Santa Clara, USA) respectively. Manufacturers’ protocols, reagents and spike-in controls were used throughout. The resultant labelled and amplified RNA (aRNA) was subject to quality control (QC) assessment of size distribution and yield using an Agilent 2100 Bioanalyzer prior to array hybridisation.

### Bioinformatic quality control and normalisation of array data

An overview of the bioinformatics pipeline is shown in Fig. 1. Expression data from 54 mRNA and 54 miRNA arrays were processed through discrete QC pipelines: mRNA via *affyAnalysisQC*^46^ and *simpleaffy*^47^; miRNA via the AgiMicroRNA package^48^. Good library quality and consistent hybridization quality were evident, however some data from both mRNA and miRNA arrays were identified as outliers and excluded from further processing (Table 1). The remaining mRNA array data (49 samples) were normalised using the GeneChip robust multiarray average (GCRMA) normalisation method^49^, while the remaining miRNA array data (44 samples) were normalised by the Robust Multi-array Average (RMA) method without correction followed by quantile normalisation^48^. Bioinformatic data analysis was performed at the Queensland Facility for Advanced Bioinformatics (QFAB).

### MicroRNA and gene expression profiling and clustering

After normalisation, preliminary filtering removed probes with coefficients of variation <0.1 across all mRNA or miRNA arrays. Next, differential expression of genes was tested using the *Limma* R package^50^ via 3 pair-wise comparisons: T vs WL; T vs NBI; WL vs NBI. Linear models were applied to the (log-transformed) mRNA and miRNA expression data, then analysed by paired *t*-tests adjusted for multiple comparisons via the Benjamini and Hochberg (BH) procedure^51^. The datasets were again filtered to remove probes that were not differentially expressed in at least one group (Padj < 0.01).

Clustering of differentially-expressed genes was achieved by Principal Component Analysis (PCA) using the *mixOmics* software package^52^. The PCA analysis used normalised expression data subject only to preliminary filtering that removed probes with coefficients of variation <0.1 across all arrays. Hierarchical clustering was performed using Cluster 3.0^53^.

### Integration of differentially expressed miRNA-mRNA

Micro-RNA sequences, annotations and chromosome positions and clustering were identified using the miRBase database (version 16)^54^. All cross-mapping of mRNA targets to miRNA effectors used the experimentally validated interactions curated in miRTarBase database (version 4.5)^22^. Our analysis sought generalised correlation models and therefore used median cohort mRNA and miRNA expression values for T, WL and NBI. MicroRNA to mRNA correlations were established using sparse partial least-square (sPLS) analysis^55^ with concurrent parameter tuning to optimise the number of mRNA retained for each miRNA. Only data for biopsies where there were both mRNA and miRNA expression data were used for integrated analysis. The statistical significance of each correlation was tested using Pearson’s product-moment correlation coefficients corrected for multiple testing using the BH procedure^51^ to calculate adjusted p-values to estimate FDR. Circos plots for visualisation of mRNA and miRNA differential expression, and correlated interactions were generated using Circa (OMGenomics.com).

### Cross validation of mRNA to miRNA correlations

Rotation estimation for each miRNA-mRNA correlation was achieved by leave one out cross validation (LOOCV) in order to establish the robustness of each putative interaction. For this, each patient’s data was omitted sequentially prior to repeating the mRNA to miRNA sPLS analysis. The number of times an mRNA target was selected (out of 18) is provided as Stability Rate, whereby a value of 1 implies perfect stability (mRNA selected 18 out of 18 times) whereas a value of 0.33 would indicate poor stability (mRNA selected 6 out of 18 times). The arbitrary threshold used here for inclusion in the final miRNA-mRNA signature was stability ≥0.5 (i.e. 50%). Circos plots for visualisation of the integrated miRNA-mRNA signature were generated using Circos Table Viewer v0.63–9^56^.

### Gene set enrichment analysis for biological pathways and gene ontology terms

Gene set enrichment analysis of biological pathways associated with differentially expressed miRNAs was performed using the miEAA tool (https://ccb-compute2.cs.uni-saarland.de/mieaa_tool/)^57^. For pathway analysis of the differentially expressed mRNA genesets we used GSEA hosted at the Broad Institute (https://genepattern.broadinstitute.org/gp/)^58^. The KEGG (Kyoto Encyclopedia of Genes and Genomes pathway) and Reactome curated pathway genesets from Molecular Signature Database (MsigDB, http://www.broadinstitute.org/gsea/msigdb/) were used. For GSEA gene differentially expressed gene lists were pre-ranked by logarithm transformation of p-value (base 10) and application of the sign of the fold-change (i.e. upregulated genes given positive values). Overrepresentation analysis (ORA) of differentially expressed genes derived from the integrated miRNA-mRNA data for pathways and gene ontology terms was performed using the web based platform EnrichR (http://amp.pharm.mssm.edu/Enrichr)^59^ which permits interrogation of multiple databases. Up and down regulated gene lists were evaluated for significant enrichment against the following gene set libraries: GO Biological Process, GO Cellular Component, GO Molecular Function (all from http://www.geneontology.org), the Wikipathways database (http://www.wikipathways.org/) and KEGG (http://www.kegg.jp/kegg) (all current as of May 2017). Enriched annotations/pathways were selected/ranked based upon combined score which was calculated by the EnrichR platform following Z-score permutation background correction on the Fischer Exact Test p-value^60^. In order to facilitate the interpretation of GO analysis the selected output annotation lists were further consolidated and summarised using REVIGO^61^, a tool that removes redundant terms based upon similarity. Network analysis was performed using Cytoscape 3.5.1^62^ and the Wikipathways plugin^63^.

## Electronic supplementary material

Supplementary Information
